# How to Spend Six Months in Paris

**Published:** 1861-11

**Authors:** 


HOW TO SPEND SIX MONTHS IN PARIS.—The Parisian correspondent of the *London Lancet*, (Sept. 28, 1861,) among other very valuable suggestions to students visiting Paris, gives the following advice :—

"1st. Dedicate two months to M. Trousseau at the Hôtel-Dieu, and Nélaton at the Clinique.

"2d. Divide one month at the Charité between Messrs. Velpeau, Beau, Nonat, and Piorry, entering with one of the latter's *internes* to a course on auscultation and percussion, the price of which for daily attendance is about thirty francs.

"3d. Give one month to St. Louis and the study of skin diseases, following Hardy in preference to either Devergie or Gibert; though, if the latter be lecturing, his course is excellent.

"4th. A month between the Ste. Eugénie and the Hôpital des Enfans Malades; at the former following Barthez and Bouchut, at the latter Guersant.

"5th. The last month to be divided between the two venereal hospitals, the Lourcine and the Midi. M. Alphonse Guérin to be followed at the former; and M. Cusco, the successor of M. Ricord, at the latter.

"During the day, the ophthalmic dispensaries of Desmarres and Sichel, and the dissecting-rooms, either of Clamart or of the École Pratique, may be attended, the cost being about £4 (100 francs) for a complete course of operative surgery and as much anatomical demonstration and dissection as the student is able to get through.

"I say nothing about attendance either upon the lectures of the Faculty, the private courses of the *internes*, or the École Pratique. I believe that it is from attendance on the hospital practice, and not from oral teaching, that the foreign student reaps most benefit. The session opens toward the middle of November."

